# Long Daytime Napping Is Associated with Increased Adiposity and Type 2 Diabetes in an Elderly Population with Metabolic Syndrome

**DOI:** 10.3390/jcm8071053

**Published:** 2019-07-19

**Authors:** Christopher Papandreou, Andrés Díaz-López, Nancy Babio, Miguel A. Martínez-González, Mónica Bulló, Dolores Corella, Montse Fitó, Dora Romaguera, Jesús Vioque, Ángel M. Alonso-Gómez, Julia Wärnberg, Alfredo J. Martínez, Lluís Serra-Majem, Ramon Estruch, José C. Fernández-García, José Lapetra, Xavier Pintó, Josep A. Tur, Antonio Garcia-Rios, Aurora Bueno-Cavanillas, Miguel Delgado-Rodríguez, Pilar Matía-Martín, Lidia Daimiel, Vicente Martín-Sánchez, Josep Vidal, Clotilde Vázquez, Emilio Ros, Pilar Buil-Cosiales, Nerea Becerra-Tomas, Raul Martinez-Lacruz, Helmut Schröder, Jadwiga Konieczna, Manoli Garcia-de-la-Hera, Anai Moreno-Rodriguez, Javier Barón-López, Napoleón Pérez-Farinós, Itziar Abete, Inmaculada Bautista-Castaño, Rosa Casas, Araceli Muñoz-Garach, José M. Santos-Lozano, Ferran Trias, Laura Gallardo-Alfaro, Miguel Ruiz-Canela, Rocio Barragan, Alberto Goday, Aina M. Galmés-Panadés, Andrés González-Botella, Jessica Vaquero-Luna, Estefanía Toledo, Olga Castañer, Jordi Salas-Salvadó

**Affiliations:** 1 Departament de Bioquímica i Biotecnologia, Unitat de Nutrició,Universitat Rovira i Virgili, 43201 Reus, Spain; 2 Institut d’Investigació Sanitària Pere Virgili (IISPV), 43201 Reus, Spain; 3 Centro de Investigación Biomédica en Red Fisiopatologia de la Obesidad y la Nutrición (CIBEROBN), Institute of Health Carlos III, 28029 Madrid, Spain; 4 Nutrition Unit, University Hospital of Sant Joan de Reus, 43201 Reus, Spain; 5 Department of Preventive Medicine and Public Health, University of Navarra, IdiSNA, 31009 Pamplona, Spain; 6 Department of Nutrition, Harvard T.H. Chan School of Public Health, Boston, MA 02115, USA; 7 Department of Preventive Medicine, University of Valencia, 46010 Valencia, Spain; 8 Cardiovascular Risk and Nutrition research group (CARIN), Hospital del Mar Research Institute (IMIM), 08003 Barcelona, Spain; 9 Clinical Epidemiology and Public Health Department, Health Research Institute of the Balearic Islands (IdISBa), 07122 Palma de Mallorca, Spain; 10 CIBER de Epidemiología y Salud Pública (CIBERESP), Instituto de Salud Carlos III, 28009 Madrid, Spain; 11 Universidad Miguel Hernández, ISABIAL-FISABIO, 03202 Alicante, Spain; 12 Department of Cardiology, Organización Sanitaria Integrada (OSI) ARABA, University Hospital Araba, 01009 Vitoria-Gasteiz, Spain; 13 Department of Nursing, School of Health Sciences, University of Málaga-IBIMA, 29016 Málaga, Spain; 14 Department of Nutrition, Food Science and Physiology, University of Navarra, IDISNA, 43204 Pamplona, Spain; 15 University of Las Palmas de Gran Canaria, Research Institute of Biomedical and Health Sciences (IUIBS), Preventive Medicine Service, Centro Hospitalario Universitario Insular Materno Infantil (CHUIMI), Canarian Health Service, 35001 Las Palmas, Spain; 16 Department of Internal Medicine, Institut d’Investigacions Biomèdiques August Pi Sunyer (IDIBAPS), Hospital Clinic, University of Barcelona, 08036 Barcelona, Spain; 17 Virgen de la Victoria Hospital, Department of Endocrinology. Instituto de Investigación Biomédica de Málaga (IBIMA), University of Málaga, 29016 Málaga, Spain; 18 Department of Family Medicine, Research Unit, Distrito Sanitario Atención Primaria Sevilla, 41013 Sevilla, Spain; 19 Lipids and Vascular Risk Unit, Internal Medicine, Hospital Universitario de Bellvitge, Hospitalet de Llobregat, 08907 Barcelona, Spain; 20 Research Group on Community Nutrition & Oxidative Stress, University of Balearic Islands & Health Research Institute of the Balearic Islands (IdISBa), 07122 Palma de Mallorca, Spain; 21 Department of Internal Medicine, Maimonides Biomedical Research Institute of Cordoba (IMIBIC), Reina Sofia University Hospital, University of Cordoba, 14071 Cordoba, Spain; 22 Department of Preventive Medicine, University of Granada, 18071 Granada, Spain; 23 Division of Preventive Medicine, Faculty of Medicine, University of Jaén, 23071 Jaén, Spain; 24 Department of Endocrinology and Nutrition, Instituto de Investigación Sanitaria Hospital Clínico San Carlos (IdISSC), 28040 Madrid, Spain; 25 Nutritional Genomics and Epigenomics Group, IMDEA Food, CEI UAM + CSIC, Madrid, Spain; 26 Institute of Biomedicine (IBIOMED), University of León, 24071 León, Spain; 27 CIBER Diabetes y enfermedades Metabólicos (CIBERDEM), Instituto de Salud Carlos III (ISCIII), 28029 Madrid, Spain; 28 Endocrinology and Nutrition Department, Hospital Clinic Universitari, 08036 Barcelona, Spain; 29 Institut d’Investigacions Biomèdiques August Pi Sunyer (IDIBAPS), 08036 Barcelona, Spain; 30 Department of Endocrinology and Nutrition, University Hospital Fundación Jimenez Díaz, 28040 Madrid, Spain; 31 Lipid Clinic, Endocrinology and Nutrition Service, Institut d’Investigacions Biomediques August Pi Sunyer (IDIBAPS), Hospital Clinic, University of Barcelona, 08007 Barcelona, Spain; 32 Department of Public Health, University of Málaga-IBIMA, 29016 Málaga, Spain; 33 Fundación para la Investigación y Prevención de Enfermedades Cardiovasculares (FIPEC), 08021 Barcelona, Spain; 34 Centro de Salud el Raval-Elx, 03203 Elche, Alicante, Spain

**Keywords:** nap, actigraphy, type 2 diabetes, body mass index, waist circumference, PREDIMED-Plus

## Abstract

Research examining associations between objectively-measured napping time and type 2 diabetes (T2D) is lacking. This study aimed to evaluate daytime napping in relation to T2D and adiposity measures in elderly individuals from the Mediterranean region. A cross-sectional analysis of baseline data from 2190 elderly participants with overweight/obesity and metabolic syndrome, in the PREDIMED-Plus trial, was carried out. Accelerometer-derived napping was measured. Prevalence ratios (PR) and 95% confidence intervals (CI) for T2D were obtained using multivariable-adjusted Cox regression with constant time. Linear regression models were fitted to examine associations of napping with body mass index (BMI) and waist circumference (WC). Participants napping ≥90 min had a higher prevalence of T2D (PR 1.37 (1.06, 1.78)) compared with those napping 5 to <30 min per day. Significant positive associations with BMI and WC were found in those participants napping ≥30 min as compared to those napping 5 to <30 min per day. The findings of this study suggest that longer daytime napping is associated with higher T2D prevalence and greater adiposity measures in an elderly Spanish population at high cardiovascular risk.

## 1. Introduction

Daytime napping (“siesta”), defined as a short sleep typically taken in the early afternoon, is a common practice in many parts of the world, including the Mediterranean region. Older adults take a nap more often than younger ones [1,2,3] and its duration can range from a few minutes to several hours. A short nap (<30 min) confers several benefits by promoting alertness, enhancing performance and improving cognitive function [4], as well as, reducing the risk of mortality [5]. On the other hand, longer naps, and especially ≥60 min/day have been associated with higher morbidity and mortality as compared with no napping [4,6]. Similarly, a higher prevalence of metabolic syndrome was observed in nappers than in non-nappers [7]. A more recent dose-response meta-analysis identified a J-curve relationship between nap time and the risk of type 2 diabetes (T2D),which is the relative risk of T2D was decreased by a short nap (<30 min), followed by a sharp increase at longer nap times [8]. However, the studies included in this meta-analysis did not use objective napping measures and therefore measurement error is inevitable. Furthermore, the association between napping and adiposity, the single most important predictor of T2D [9], has only scarcely been investigated. A previous prospective study evaluating associations between subjective measures of nap and the incidence of obesity suggested that napping for 30 min per day may be a protective factor for obesity [10]. On the other hand, each one hour increase in objectively-measured daytime napping was associated with greater adiposity in the Study of Osteoporotic Fractures [11] and, recently, in the Hispanic Community Health Study/Study of Latinos [12]. To the best of our knowledge, no study has previously examined associations between objectively-measured napping time and T2D. Furthermore, daytime napping in relation to T2D and adiposity measures has been mostly examined in non-Mediterranean populations [8]. Whether there are differences in the health effects of daytime napping between Mediterranean countries where nap time is considered as a common tradition and countries where nap is not a routine is unknown. Furthermore, examining the relationship between nap and T2D in older adults with metabolic syndrome is of public health importance for the development of interventions specifically targeted at the improvement of sleep habits in this vulnerable group.

Therefore, in the present cross-sectional study based on the PREDIMED-Plus trial, we addressed the following hypotheses: Longer daytime napping is associated with (1) a high prevalence of T2D, and (2) greater body mass index (BMI) and waist circumference (WC).

## 2. Methods

### 2.1. Study Design and Sample

**Figure 1:**
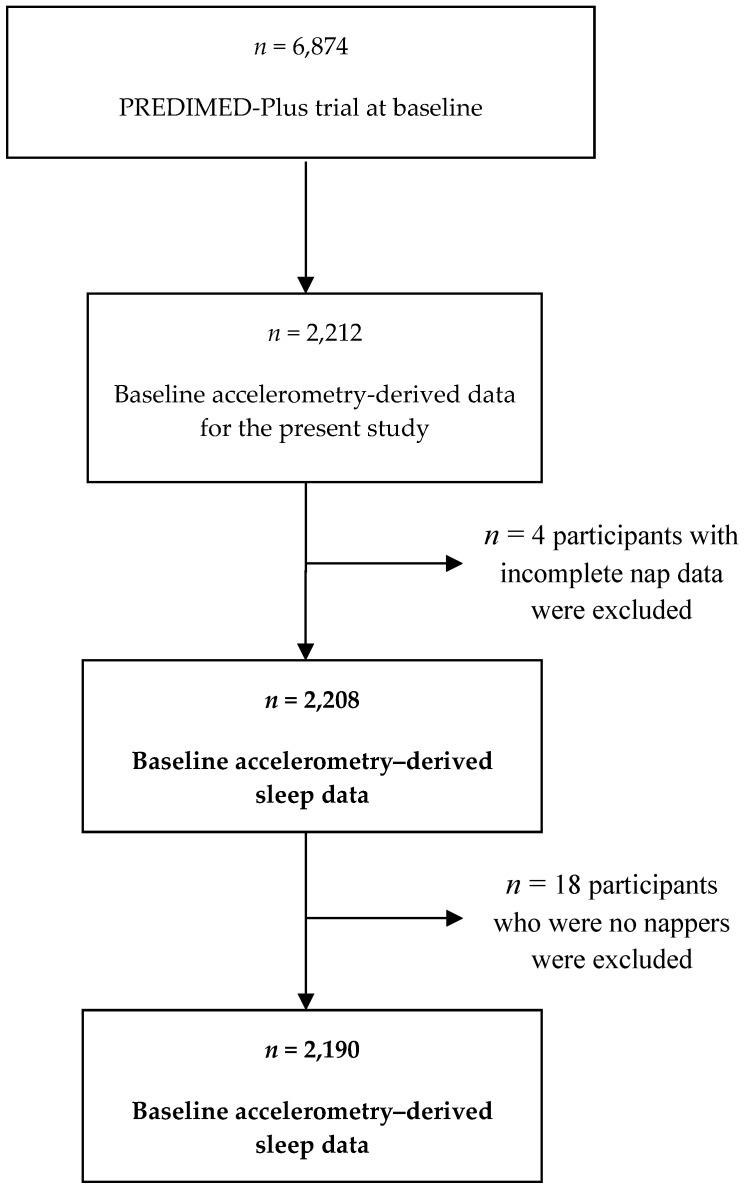
Flow-chart of study participants.

We cross-sectionally analyzed baseline data from the PREDIMED-Plus trial, a 6-year ongoing parallel-group, multicenter lifestyle intervention study involving 6,874 participants from Spain. The design of the PREDIMED-Plus trial has been described in detail elsewhere [13]. Community-dwelling adults (ages 55–75 years) with BMI ≥ 27 and <40 kg/m^2^, and meeting ≥3 metabolic syndrome individual components were included [14]. Out of the 6,874 participants, data derived from accelerometry was available in a subsample of 2,190 participants, while 4 participants were excluded due to incomplete nap data and an additional 18 participants who were no nappers (Figure 1). Therefore, the final sample size for the present analysis was 2205. The institutional review boards of the recruitment centers approved the study protocol, and participants provided written informed consent.

### 2.2. Sleep Assessment by Accelerometry

A wrist-worn triaxial accelerometer (GENEActiv, ActivInsights Ltd., Kimbolton, UK) was provided to participants who were asked to wear it on their non-dominant wrist for 8 consecutive 24-h days. At least 2 valid days and ≥16 h per day of accelerometer usage were considered for the present analyses. The data was recorded at 40 Hz with a ±8 g dynamic range, and acceleration data was expressed relative to gravity (g) units (1 g = 9.81 m/s^2^). Raw data were processed using the open-source R-package GGIR, version 1.7-1 [15,16]. Daytime napping was estimated as a period of sustained inactivity during the day, itself detected as the absence of change in arm angle greater than 5 degrees for at least 5 min [17]. The average sleep duration was also calculated [17].

### 2.3. T2D Prevalence

T2D was defined as previous clinical diagnosis of diabetes, or HbA1c ≥6.5% or use of antidiabetic medication or insulin at baseline or fasting plasma glucose >126 mg/dL in both the screening visit and baseline visit [18].

### 2.4. Adiposity Measures and Other Covariates

Weight and height were measured with light clothing and no shoes with calibrated scales and a wall-mounted stadiometer, respectively. BMI was calculated as weight (kg) divided by height (meters) squared. WC (cm) was measured half way between the last rib and the iliac crest by using an anthropometric tape. All anthropometric variables were determined in duplicate. Information about age, sex, education, marital and employment status, smoking, depression, sleep apnea and use of sedatives were provided by structured interviews. Adherence to an energy-restricted Mediterranean diet (MedDiet) was assessed using a 17-item questionnaire. Physical activity was measured using the accelerometer and moderate to vigorous physical activity (MVPA) was calculated as described previously [19]. A new binary variable was created according to compliance of the WHO recommendations for MVPA set in ≥150 min/week [20].

### 2.5. Statistical Analysis

The normal distribution of the variables was evaluated using the Kolmogorov-Smirnov test. Participants’ characteristics according to categories of daytime napping are presented as means (±SD) or median (interquartile range) for quantitative variables, and percentages (%) and numbers (*n*) for categorical variables. These characteristics were examined across four categories of daytime napping (5 to <30, 30 to <60, 60 to <90, and ≥90 min). One-way ANOVA (Bonferroni post hoc analysis for pairwise comparisons) or Kruskal-Wallis (Mann-Whitney tests in the post-hoc multiple comparisons) and Chi-square tests were used, as appropriate, to assess differences in characteristics according to nap categories. Since T2D is highly prevalent (>10%), Cox regression models with constant time of follow-up set at *t* = 1 (given the cross-sectional design) and robust variance estimates [21,22] were applied to assess prevalence ratios (PR) for T2D. We compared three categories of napping (30 to <60, 60 to <90, and ≥90 min) for prevalent T2D using those participants napping between 5 and <30 min as the reference category based on reports [4,5]. We also examined the associations of 10 ln transformed minutes increment in daytime napping with T2D. Three models were examined. Model 1 was adjusted for sex and age (continuous). Model 2 was further adjusted for BMI (continuous), marital status (single/divorced, married and widower), employment (working, nonworking, retired), education (primary education, secondary education, academic/graduate), smoking habit (current smoker, past smoker, never smoked), sedative treatment (yes/no), sleep apnea (yes/no), hypertension (yes/no), depression (yes/no), 17-score energy-restricted Mediterranean diet (continuous), compliance to MVPA recommendations (yes/no), sleep duration(continuous) and intervention center. A third model was fitted by including variables from model 2 except for BMI which was replaced by WC. Linear regression models were also fitted to compare the three categories of napping with the reference category for BMI and WC measures. We also examined the associations of 10 ln transformed minutes increment in daytime napping with BMI and WC. For these analyses we used models 1 and 2 but instead of obesity we adjusted for T2D (yes/no). Prior to usage of daytime napping as continuous variable a log-transformation was performed. We also added a multiplicative term (1 degree of freedom) between education status (secondary education, academic/graduate merged versus primary education) and categories of daytime napping into the multivariable regression models for T2D, BMI and WC stratified on education status, to test for interactions by means of likelihood ratio tests. The low number of current smokers (11%) and current workers (20%) did not allow us to further test for interactions and stratify analyses by these variables. Significance was set at p values <0.05. All analyses were performed using STATA, version 14.1 (StataCorp. LP, College Station, TX, USA) using the available 12 March 2019 PREDIMED-Plus database.

## 3. Results

General characteristics for the total population and by categories of daytime napping are displayed in Table 1. The mean age of the participants was 65.0 years and the median napping was 62.4 (39.0; 92.4) with a range from 5.4 to 275.4 min per day. According to napping categories, those participants napping 5 to <30 min per day were more likely to be young and women, adhere more to the MedDiet and MVPA recommendations, and less likely to be current smokers. They were also more likely to have lower adiposity measures and T2D and be currently employed. In addition, increased napping was associated with reduced sleep duration. No significant differences in main characteristics such as age, sex, weight, BMI, WC, obesity, and T2D prevalence rates were found between participants with accelerometry-derived sleep data and the rest of the participants enrolled in the PREDIMED-Plus trial. Furthermore, non-nappers had no significant differences in metabolic characteristics compared with nappers.

In the fully adjusted model, 10 ln transformed minutes increment daytime napping was associated with a higher prevalence of T2D (PR 1.17 (1.04, 1.32), *p* = 0.008) (Table 2). Furthermore, compared with those napping 5 to <30 min per day, participants napping ≥90 min, had a higher prevalence of T2D (PR 1.40 (1.08, 1.82), *p* = 0.011) (Table 2). Further adjustment for WC instead of BMI slightly changed the results.

Table 3 shows the β-coefficients (95% CI) for BMI and WC per 10 ln transformed minutes/day increment in daytime napping and according to napping categories. In the fully adjusted model, 10 ln transformed minutes/day increment napping was directly associated with BMI and WC ((β = 0.48, *p* < 0.001) and (β = 1.04, *p* < 0.001), respectively). Participants napping between 30 and 60 min, between 60 and 90 min, and ≥90 min per day had increased BMI, as compared to those napping 5 to <30 min ((β = 0.73, *p* = 0.001), (β = 0.84, *p* ≤ < 0.001) and (β = 1.11, *p* < 0.001), respectively). Additionally, participants napping between 30 and 60 min, between 60 and 90 min, and at least 90 min per day had increased WC, as compared to those napping 5 to <30 ((β = 1.52, *p* = 0.009), (β = 1.51, *p* = 0.012) and (β = 2.43, *p* < 0.001), respectively). In stratified analyses by education status, low educated participants napping between 30 and 60 min, between 60 and 90 min, and ≥90 min per day had increased BMI, as compared to those napping 5 to <30 min ((β = 0.69, *p* = 0.036), (β = 1.08, *p* = 0.002) and (β = 1.55, *p* < 0.001), respectively). On the other hand, high educated participants napping between 30 and 60 min, between 60 and 90 min, and ≥90 min per day had increased BMI, as compared to those napping 5 to <30 min ((β = 0.87, *p* = 0.004), (β = 0.72, *p* = 0.022) and (β = 0.70, *p* = 0.030), respectively), (*p* for interaction = 0.007).

## 4. Discussion

In the present cross-sectional study of 2,190 elderly participants from the PREDIMED-Plus trial, we observed that 10 ln transformed minutes increase in daytime napping was associated with a higher prevalence of T2D and greater BMI and WC. Furthermore, compared to napping 5 to <30 min, napping ≥90 min per day was associated with higher T2D prevalence, whereas napping at least 30 min per day was positively associated with adiposity measures.

To the best of our knowledge, this is the first study examining associations between objectively measured napping and T2D. A previous meta-analysis of case-control studies, including elderly adults, showed that a longer self-reported nap time (≥60 min/day) was associated with higher odds for T2D versus no nap, while napping for 90 min increased the risk by up to 50% [8]. Similarly, in our study, the magnitude of the association with T2D increased when participants napped for at least 90 min and was independent of potential confounders including sleep duration, diet, physical activity, and adiposity. Potential disturbances in the immune–endocrine system could explain the aforementioned association. Increased sympathetic activity upon awaking from daytime naps, especially prolonged ones, could lead to disruption of the sympatho-vagal balance, activation of the renin-angiotensin system, and subsequent decrease in pancreatic beta-cell secretion and glucose dysregulation [23,24]. Increased sympathetic nervous system activity could also induce elevations in cortisol levels. Post-nap cortisol increases have been observed following 90-min afternoon napping compared to 50-min ones [25]. Whether longer napping increased cortisol levels among our study participants leading to glucometabolic disturbances, including insulin resistance, and eventually T2D, is a hypothesis that deserves further investigation [26]. Furthermore, daytime napping has been associated with higher levels of IL-6 [27] and C-reactive protein [28]. Since these pro-inflammatory markers have been associated with increased risk ofT2D [29], this could be another plausible explanation. Given the cross-sectional nature of this study, we cannot exclude the possibility that daytime napping could be a consequence of T2D. Indeed, in the Health ABC study diabetic patients had higher odds of day time napping compared to those without T2D [30].

The associations between napping and adiposity measures are in line with previous studies [10,11] and independent of sleep duration, diet, physical activity, and diabetes status. Napping ≥30 min per day may have induced elevations in post-nap cortisol levels which, in turn, may increase fat deposition [31] and induce alterations in appetite with a preference for more palatable, energy-dense foods [32]. These observations are suggestive of a causal role of cortisol in the development of greater adiposity. It is of interest that the magnitude of these associations was higher for WC than BMI. Taking this into account, we can speculate that cortisol elevations following napping may lead to the accumulation of fat in the abdominal area through fat redistribution from peripheral to central depots [26]. Inflammation triggered by napping may also result in increments in adiposity. In two longitudinal studies, a large weight gain was more common in subjects with elevated markers of inflammation [33,34].

It would be important to mention that previous studies [7,8] evaluating self-report or objective napping in non-Mediterranean populations found higher prevalence of non-napping than in our study. Even in studies recruiting participants from the Mediterranean region, the prevalence of non-nappers was notably higher [5,10]. Our specific aged population with metabolic syndrome and errors in the interpretation of napping due to the methodology (lack of sleep log including specific questions for naps) could explain these discrepancies.

## 5. Limitations

A limitation of the study is its cross-sectional design, which does not allow any causal inference of the observed associations to be made. Daytime napping might be the result of the high prevalence of metabolic syndrome affecting the quality of night sleeping. The study participants across higher categories of napping appeared to sleep less in the night and this may be due to other concomitant diseases accompanying the metabolic syndrome. Another limitation is that the participants were elderly Mediterranean patients with metabolic syndrome. Thus, results cannot be extrapolated to other ethnicities, age groups, and subjects without metabolic syndrome. Furthermore, we cannot rule out the possibility of residual and unmeasured confounding such as family history of T2D, socioeconomic status, insomnia and daytime sleepiness. In addition, even though we adjusted for sleep duration; sleep quality may confound the results, but given the strong statistical significance, it seems unlikely that a nocturnal sleep pattern entirely explains these associations. The lack of non-nappers in our sample also does not allow us to conclude the association between short naps and T2D as relative to no-naps. Nap frequency was also not analysed in our study, which could shed light on the complicated relationships with T2D and adiposity. Finally, we did not use a sleep log including specific questions for naps and it is possible that the association between napping and outcomes of interest may actually reflect periods of quiet wakefulness.

## 6. Conclusions

In conclusion, longer napping was associated with higher prevalent T2D and greater adiposity measures in an elderly Spanish population at high cardiovascular risk. Whether cortisol and inflammation could contribute to the relationship between napping, T2D, and increased adiposity is a hypothesis that deserves further investigation. More prospective studies are needed to confirm our findings, clarify the temporal nature of this relationship, and elucidate possible mechanisms underlying these observations.

## Figures and Tables

**Table 1:** Baseline characteristics of the study population from PREDIMED-Plus trial across categories of daytime napping.

			Categories of Daytime Napping (min)				
		Total *n* = 2190	5 to <30 min *n* = 344	30 to <60 min *n* = 692	60 to <90 min *n* = 562	≥90 min *n* = 592	*P*-Value
**Sleep parameters**							
	Napping duration, median interquartile range, min	62.4 (39.0; 92.4)	20.4 (14.4; 25.2)	44.4 (37.2;52.2)	73.2 (66.6;79.8)	115.8 (100.8; 139.2)	<0.001
	Napping duration, min, max	5.4, 275.4	5.4, 29.4	30.0, 59.4	60.0, 89.4	90.0, 275.4	
	Sleep duration, mean ± SD, h	6.2 ± 1.2	6.4 ± 1.0	6.3 ± 1.0	6.1 ± 1.2	5.9 ± 1.5	<0.001
**Age, mean ± SD, y**		65 ± 5	64 ± 5	65 ± 5	65 ± 5	65 ± 5	0.001
**Male, *n* (%)**		1163(53)	145(40)	361(52)	300(53)	362(61)	<0.001
**BMI, mean ± SD, kg/m^2^**		32.6 ± 3.4	32.0 ± 3.3	32.6 ± 3.3	32.7 ± 3.5	32.9 ± 3.5	0.001
**WC, mean ± SD, cm**		107.4 ± 9.5	104.8 ± 9.4	107.2 ± 9.4	107.4 ± 9.4	109.1 ± 9.6	<0.001
**Type 2 diabetes, *n* (%)**		720(33)	89(24)	216(31)	191(34)	228(38)	<0.001
**Sleep apnea, *n* (%)**		283(13)	47(13)	85(12)	74(13)	79(13)	0.944
**Depression, *n* (%)**		482(22)	70(19)	140(20)	125(22)	152(25)	0.049
**Sedative treatment, *n* (%)**		530(24)	90(25)	153(22)	138(24)	156(26)	0.365
**Smoking, *n* (%)**							
	Never	940(43)	195(54)	317(46)	230(41)	207(35)	<0.001
	Former	989(45)	141(39)	309(45)	270(48)	277(47)	
	Current	253(11)	26(7)	62(9)	59(10)	107(18)	
**Adherence to energy-restricted MedDiet** **(score from 0 to 17 item), mean ± SD**		8.5 ± 2.7	8.9 ± 2.7	8.4 ± 2.6	8.5 ± 2.9	8.6 ± 2.6	0.083
**Compliance of MVPA recommendations ^a^, *n* (%)**		755(34)	130(36)	252(36)	211(37)	169(28)	0.005
**Education status, *n* (%)**							
	Primary education	1071(49)	174(48)	341(49)	265(47)	303(52)	0.088
	Secondary education	610(28)	107(30)	203(29)	147(26)	158(27)	
	Academic/graduate	479(22)	78(21)	141(20)	139(25)	122(21)	
**Employment status, *n* (%)**							
	Working	439(20)	93(26)	127(21)	115(21)	86(15)	<0.001
	Non-working	535(24)	102(28)	197(28)	121(21)	135(23)	
	Retired	1215(55)	165(45)	365(53)	322(58)	365(62)	
**Marital status, *n* (%)**							
	Single/divorced	323(14)	62(17)	83(12)	84(15)	94(16)	0.716
	Married	1630(75)	262(72)	536(77)	415(74)	432(73)	
	Widower	228(12)	37(10)	72(10)	59(10)	62(10)	

Data are presented as mean ± SD or median interquartile range unless otherwise indicated. Abbreviations: BMI, body mass index; WC, waist circumference; MedDiet, Mediterranean Diet; MVPA, moderate to vigorous physical activity. ^a^ Recommendations for MVPA set on ≥150 min/week for elderly persons, based on accelerometry-derived 10-min bout MVPA. *P*-value for differences between categories of nocturnal sleep duration was calculated by chi-square or one-way analysis of variance test for categorical and continuous variables, respectively. In case of non-normally distributed variables we performed Kruskal-Wallis test.

**Table 2:** Multivariable-Prevalence Ratio (95% CI) for type 2 diabetes according to categories of daytime napping and per increment of 10 min in daytime napping (ln-transformed).

	Categories of Daytime Napping (min)				10 min Increment in Daytime Napping (ln Transformed)				
	5 to <30 min	30 to <60 min	60 to <90 min	≥90 min	*P*-Value 2 vs. 1	*P*-Value 3 vs. 1	*P*-Value 4 vs. 1	Continuous	*P*-Value
n	344	692	562	592				2190	
T2D (%) n	(24) 89	(31) 216	(34) 191	(38) 228					
**Model 1**	1 (ref.)	1.24 (0.96, 1.59)	1.34 (1.04, 1.74)	1.50 (1.17, 1.93)	0.096	0.025	0.002	1.22 (1.09, 1.37)	0.001
**Model 2**	1 (ref.)	1.25 (0.97, 1.61)	1.29 (0.99, 1.68)	1.40 (1.08, 1.82)	0.086	0.054	0.011	1.17 (1.04, 1.32)	0.008
**Model 3**	1 (ref.)	1.24 (0.96, 1.60)	1.28 (0.98, 1.66)	1.37 (1.06, 1.78)	0.101	0.064	0.017	1.16 (1.03, 1.31)	0.012

Model 1 adjusted for sex, age (years). Model 2 adjusted for Model 1 plus body mass index, marital status (single/divorced, married and widower), employment (working, nonworking, retired), education (primary education, secondary education, academic/graduate), smoking habit (current smoker, past smoker, never smoked), sedative treatment (yes/no), sleep apnea (yes/no), hypertension (yes/no), depression (yes/no), 17-score energy-restricted Mediterranean diet, compliance to MVPA recommendations set in ≥150 min/week (yes/no), sleep duration and intervention center. Model 3 adjusted for Model 2 except for body mass index that was replaced by waist circumference. Category 1 Participants napping 5 to <30 min per day. Category 2 Participants napping between 30 and 60 min per day. Category 3 Participants napping between 60 and 90 min per day. Category 4 Participants napping ≥90 min per day.

**Table 3:** Multivariable-adjusted β-coefficients (95% CI) according to categories of daytime napping and per increment of 10 min in daytime napping (ln-transformed) in relation to body mass index and waist circumference.

	Categories of Daytime Napping (min)				10 min Increment in Daytime Napping (ln Transformed)				
	5 to <30 min	30 to <60 min	60 to <90 min	≥90 min	*P*-Value 2 vs. 1	*P*-Value 3 vs. 1	*P*-Value 4 vs. 1	Continuous	*P*-Value
**BMI, kg/m^2^**									
n	344	692	562	592				2190	
**Model 1**	0 (ref.)	0.70 (0.26, 1.15)	0.82 (0.35, 1.28)	1.15 (0.69, 1.61)	0.002	0.001	<0.001	0.50 (0.28, 0.71)	<0.001
**Model 2**	0 (ref.)	0.73 (0.29, 1.16)	0.84 (0.38, 1.29)	1.11 (0.65, 1.58)	0.001	<0.001	<0.001	0.48 (0.27, 0.70)	<0.001
**WC, cm**									
n	344	692	562	592				2190	
**Model 1**	0 (ref.)	1.66 (0.50, 2.81)	1.75 (0.55, 2.95)	2.95 (1.75, 4.15)	0.005	0.004	<0.001	1.31 (0.75, 1.86)	<0.001
**Model 2**	0 (ref.)	1.52 (0.39, 2.66)	1.51 (0.33, 2.70)	2.43 (1.23, 3.64)	0.009	0.012	<0.001	1.04 (0.47, 1.60)	<0.001

BMI, body mass index; WC, waist circumference. Model 1 adjusted for sex, age (years). Model 2 adjusted for Model 1 plus type 2 diabetes (yes/no), marital status (single/divorced, married and widower), employment (working, nonworking, retired), education (primary education, secondary education, academic/graduate), smoking habit (current smoker, past smoker, never smoked), sedative treatment (yes/no), sleep apnea (yes/no), hypertension (yes/no), depression (yes/no), 17-score energy-restricted Mediterranean diet, compliance to MVPA recommendations set in ≥150 min/week (yes/no), sleep duration and intervention center. Category 1 Participants napping 5 to <30 min per day. Category 2 Participants napping between 30 and 60 min per day. Category 3 Participants napping between 60 and 90 min per day. Category 4 Participants napping ≥90 min per day.

## Data Availability

The datasets generated and analysed during the current study are not publicly available due to national data regulations and for ethical reasons, including the possibility that some information might compromise research participants’ consent because our participants only gave their consent for the use of their data by the original team of investigators. However, these data can be requested by signing a data sharing agreement as approved by the relevant research ethics committees and the steering committee of the PREDIMED-Plus study.
